# A multi-site resting state fMRI study on the amplitude of low frequency fluctuations in schizophrenia

**DOI:** 10.3389/fnins.2013.00137

**Published:** 2013-08-08

**Authors:** Jessica A. Turner, Eswar Damaraju, Theo G. M. van Erp, Daniel H. Mathalon, Judith M. Ford, James Voyvodic, Bryon A. Mueller, Aysenil Belger, Juan Bustillo, Sarah McEwen, Steven G. Potkin, FBIRN, Vince D. Calhoun

**Affiliations:** ^1^Mind Research NetworkAlbuquerque, NM, USA; ^2^Department of Psychiatry, University of New MexicoAlbuquerque, NM, USA; ^3^Department of Psychiatry and Human Behavior, University of California IrvineIrvine, CA, USA; ^4^Department of Psychiatry, University of California, San FranciscoSan Francisco, CA, USA; ^5^San Francisco VA Medical CenterSan Francisco, CA, USA; ^6^Department of Radiology, Brain Imaging and Analysis Center, Duke UniversityDurham, NC, USA; ^7^Department of Psychiatry, University of MinnesotaMinneapolis, MN, USA; ^8^Department of Psychiatry, University of North Carolina School of MedicineChapel Hill, NC, USA; ^9^Department of Psychiatry and Biobehavioral Sciences, University of CaliforniaLos Angeles, Los Angeles, CA, USA; ^10^Electrical Engineering, University of New MexicoAlbuquerque, NM, USA

**Keywords:** resting state fMRI, LFO, ALFF, schizophrenia, multi-site studies, effect size

## Abstract

**Background:** This multi-site study compares resting state fMRI amplitude of low frequency fluctuations (ALFF) and fractional ALFF (fALFF) between patients with schizophrenia (SZ) and healthy controls (HC).

**Methods:** Eyes-closed resting fMRI scans (5:38 min; *n* = 306, 146 SZ) were collected from 6 Siemens 3T scanners and one GE 3T scanner. Imaging data were pre-processed using an SPM pipeline. Power in the low frequency band (0.01–0.08 Hz) was calculated both for the original pre-processed data as well as for the pre-processed data after regressing out the six rigid-body motion parameters, mean white matter (WM) and cerebral spinal fluid (CSF) signals. Both original and regressed ALFF and fALFF measures were modeled with site, diagnosis, age, and diagnosis × age interactions.

**Results:** Regressing out motion and non-gray matter signals significantly decreased fALFF throughout the brain as well as ALFF in the cortical edge, but significantly increased ALFF in subcortical regions. Regression had little effect on site, age, and diagnosis effects on ALFF, other than to reduce diagnosis effects in subcortical regions. There were significant effects of site across the brain in all the analyses, largely due to vendor differences. HC showed greater ALFF in the occipital, posterior parietal, and superior temporal lobe, while SZ showed smaller clusters of greater ALFF in the frontal and temporal/insular regions as well as in the caudate, putamen, and hippocampus. HC showed greater fALFF compared with SZ in all regions, though subcortical differences were only significant for original fALFF.

**Conclusions:** SZ show greater eyes-closed resting state low frequency power in frontal cortex, and less power in posterior lobes than do HC; fALFF, however, is lower in SZ than HC throughout the cortex. These effects are robust to multi-site variability. Regressing out physiological noise signals significantly affects both total and fALFF measures, but does not affect the pattern of case/control differences.

## Introduction

Resting state fMRI has numerous advantages over other neuroimaging techniques to elucidate the pathopysiology of disease states. Compared to other neuroimaging methods resting state fMRI is non-invasive and does not expose subjects to radiation (i.e., PET), has higher spatial resolution (compared to EEG), and is easily applied in almost all clinical populations since it does not require participation in a cognitive task (i.e., task-driven fMRI). The low-frequency fluctuations within resting state fMRI signals are considered to reflect spontaneous neuronal activity to some degree [for one review of this issue, see van den Heuvel and Hulshoff Pol (2010)]. Within resting state fMRI research there are many analysis techniques to apply to determine areas of spontaneous coherent BOLD signal (and by hypothesis, correlated neural activity) across brain regions. Seed-based connectivity and multivariate decompositions such as independent component analysis (ICA) can identify brain areas that have related BOLD signal time courses. These methods can be used both to characterize healthy brain function as well as dysfunction in clinical populations. Resting state fMRI studies in schizophrenia (SZ) have used these methods to examine connectivity between the cortex and subcortical structures (Welsh et al., 2010), the differences in cortical networks (Liang et al., 2006; Jafri et al., 2008; Woodward et al., 2011), the relationship between structure and function (Michael et al., 2010), the relationships between various resting state measures and cognitive performance (He et al., 2012; Meier et al., 2012; Zou et al., 2012), as well as assessing the graph-theoretic organization (Bassett et al., 2008; Yu et al., 2011; Ma et al., 2012).

Besides temporal correlations, we can also analyze the within-voxel time course and consider its power across the range of spatial frequencies (Biswal et al., 1997; Yang et al., 2007; Zuo et al., 2010). The amplitude of low frequency fluctuations (ALFF) for a voxel's timecourse is the calculated power in the very low frequencies, usually 0.01–0.08 Hz. Most often this measure is scaled by the subject's mean ALFF value across voxels, analogous to PET analyses (Zang et al., 2007). A second variation is to examine ALFF as a fraction of the observed power in all available frequencies, or fractional ALFF (fALFF) (Zou et al., 2008). The first measure captures low frequency power relative to the mean low frequency power across voxels, thus indicating spatial variations relative to the mean; the second measure expresses low frequency power relative to the overall power in the same voxels, and as such may keep the large vessel fluctuations localized rather than spreading across all voxels. These measures assess a regional homogeneity of resting state activity, and are commonly stronger in the default mode network areas (DMN) (Zang et al., 2007). There is growing evidence that they relate to cortical excitability and long-range neural synchronization (Buzsaki and Draguhn, 2004; Balduzzi et al., 2008; Zuo et al., 2010; Di et al., 2013). Zuo et al. measured ALFF and fALFF on a sample of healthy subjects and showed that the two measures are highly related, but not entirely the same; both were largest in gray matter, particularly in the occipital and posterior cingulate/precuneus cortex, and both showed good to moderate test-retest reliability (Zuo et al., 2010). ALFF and fALFF measurements also have been associated with task performance (Mennes et al., 2011; Zou et al., 2012), default-mode activity between active blocks (Zhang and Li, 2012), and in some cases with inter-trial variability in BOLD signal (Liu et al., 2011).

The use of resting state ALFF measurements and fALFF measurements have been applied to studies of chronic and first episode SZ, with mixed results. Repeated measurements of ALFF with up to a year between scans in healthy controls (HC) and chronic, stable SZ patients have been shown to be highly repeatable (Turner et al., 2012). Studies focusing on first episode patients have identified decreased ALFF in the ventromedial prefrontal cortex, and increased ALFF in the left and right putamen (Huang et al., 2010; Lui et al., 2010). In long-term SZ, one study of resting state ALFF and fALFF reported lower ALFF and fALFF in the primary sensory areas in patients, higher ALFF and fALFF in patients in the hippocampus, but higher fALFF only in medial prefrontal cortex (Hoptman et al., 2010). Yu et al. considered spectral power in chronic SZ across several frequency bands spanning 0.01–0.25 Hz, and confirmed clusters of ALFF decreases in the middle occipital lobe, precuneus, parietal lobule, and the insula (Yu et al., 2012). They also identified ALFF increases in the inferior frontal, medial frontal gyri, and middle temporal gyri, in agreement with Hoptman's results but not Huang's. fALFF results showed decreased fALFF for patients in the pre and postcentral gyri, fusiform and lingual gyri, and increased fALFF in the insula, parietal lobe, and medial frontal gyrus (Yu et al., 2012). The current literature supports the hypothesis that areas of decreased ALFF and fALFF in patients with SZ are in the posterior brain, while increases, if they exist, are more anterior. Where in particular the increases are, and whether decreases exist in the frontal lobe, has varied by study.

Within the ALFF methodology, some studies have regressed the head movement parameters out of each subject's time course prior to computing ALFF on the residuals (Li et al., 2012), while others have not (Yang et al., 2007). Similarly, the question exists of whether the mean signal from non-gray matter sources such as white matter (WM) or cerebral spinal fluid (CSF) should be removed from resting state data prior to these analyses. Regressing out head movement and physiological signals from WM and CSF have been recommended for resting state fMRI analyses to remove artefactual effects (Birn, 2012) but with a focus on connectivity measures, rather than low frequency power. A recent massive study of processing choices for standardizing resting state data across analysis techniques indicated that head movement does affect individual ALFF measures though not fALFF, and regressing global signal will affect ALFF (Yan et al., 2013). What effect these image processing methods have on identifying the differences across disease states, however, has not been explored.

The Functional Imaging Biomedical Informatics Research Network (FBIRN) developed methods for multi-site fMRI studies in clinical populations, notably SZ (Glover et al., 2012). The FBIRN Phase III study includes resting state fMRI data from patients with SZ and HC across seven different universities. We used the data from this large multi-site study for two purposes: First, to assess in a larger sample what the regional differences are in ALFF and fALFF between patients with SZ and HC, and whether they are reliable across sites; and second, whether these differences are affected by the choice to regress out motion, brain CSF and WM-based physiological noise. We also evaluate the variability across the data collection sites, to provide a sense of the expected variability in published results across studies of independent samples in these measures.

## Materials and methods

### Subjects

The resting state fMRI data presented here was collected on 186 SZ patients (mean age ± SD = 38.9 ± 11.6, 145 males) and 176 healthy volunteers (mean age ± SD = 37.5 ± 11.2, 126 males) matched as much as possible for age, sex, handedness, and race distributions, recruited from seven sites, who participated in the study. Inclusion criteria for the patients were a SZ diagnosis based on the Structured Clinical Interview for DSM-IV-TR Axis I Disorders (SCID-I/P) (First et al., 2002b). All patients were clinically stable on antipsychotic medication for at least 2 months, and had an illness duration of minimally 1 year. SZ patients and healthy subjects were excluded if they had a history of major medical illness, contraindications for MRI, eyesight that could not be corrected to normal acuity with MRI compatible corrective lenses, a history of drug dependence in the last 5 years or a current substance abuse disorder (except for nicotine), or an IQ less than 75. Patients with clinically significant tardive dyskinesia and healthy subjects with a current or past history of major neurological or psychiatric illness (SCID-I/NP) (First et al., 2002a) or with a first-degree relative with an Axis-I psychotic disorder diagnosis were also excluded. In addition to the SCID-I/P, the clinical assessments for the patients included the Positive and Negative Syndrome Scale (PANSS) (Kay et al., 1989). Both patients and controls were assessed with the Hollingstead Socioeconomic Status Scale (HSSS) (Hollingstead, 1975), and a basic demographics form, and other assessments not reported here. Clinicians at each of the centers participated in training sessions to calibrate clinical ratings.

Written informed consent was obtained from all study participants, and included permission to share de-identified data between the centers and with the wider research community. The consent process was approved by the University of California Irvine, the University of California Los Angeles, the University of California San Francisco, Duke University, University of North Carolina, University of New Mexico, University of Iowa, University of Minnesota Institutional Review Boards.

### MRI data acquisition

The resting state fMRI data were collected as part of a larger experimental protocol, including memory task-based fMRI, arterial spin labeling (ASL), and diffusion tensor imaging (DTI) scans. We report here only on the resting state fMRI scans, which were collected for all subjects approximately halfway through the scanning session, following the structural and ASL scans, and half of the memory task scans.

The seven sites included six 3T Siemens TIM Trio scanners and one 3T MR750 GE scanner (coded as the first site in all following graphs). The imaging protocol for the resting state scans at all sites was a T2^*^-weighted AC-PC aligned echo planar imaging sequence (TR/TE 2 s/30 ms, flip angle 77 degrees, 32 slices collected sequentially from superior to inferior, 3.4 × 3.4 × 4 mm with 1 mm gap, 162 frames, 5:38 min). For the resting scan, subjects were instructed to lie still with eyes closed.

### Image processing methods

#### Quality assurance

The three translation and three rotational head movement parameters for each subject were checked for maximal overall movement relative to the first image. Subjects who moved more than 4 mm were excluded from the analysis. We additionally used a data driven criterion to select subjects for the full analysis. We computed signal fluctuation to noise ratio (SFNR) (Friedman et al., 2006) defined as the ratio of mean signal intensity across time and space to the average standard deviation of the same voxel time series in a ROI in the center of brain for every subject, using *dataQuality* matlab package (http://cbi.nyu.edu/software/dataQuality.php). The SFNR for each subject was plotted against maximal absolute motion and average frame to frame motion; subjects with SFNR less than 150 tended also to have greater average frame to frame motion. Thus we excluded subjects who had a SFNR less than 150, or who moved more than 4 mm total.

#### Preprocessing

The images were preprocessed using the MRN automated analysis pipeline (Bockholt et al., 2010), whose steps are conducted in SPM 5 (http://www.fil.ion.ucl.ac.uk/spm) as follows: Motion correction to the first image using INRIalign; slice timing corrected to the middle slice; and normalization to MNI space, including reslicing to 3 × 3 × 3 mm voxels. These normalized images were the input to the original ALFF analysis. For the regressed analyses prior to ALFF or fALFF calculations and smoothing, we orthogonalized each within-brain voxel's time series with respect to the mean time series from the subject's WM, CSF signals, and the six head motion parameters corresponding to the subject as well as linear and quadratic trends,. To compute WM and CSF masks, we obtained WM and CSF segmentations from each subject's high resolution structural scans using SPM's VBM framework. Average WM and CSF segmentations across all subjects were computed in MNI space. These mean segmentation maps were thresholded at a very conservative level (*T* > 0.99) to reduce cross-contamination from other tissue types, and binarized. These binarized maps were resampled to EPI resolution using nearest neighbor interpolation. Average timeseries from these WM and CSF ROIs have been shown to correspond to physiological noise related to breathing and cardiac pulsations (Lund and Hanson, 2001).

#### ALFF and fALFF calculation

ALFF images were computed in a similar way to the REST software (http://resting-fmri.sourceforge.net) (Song et al., 2011), through linearly detrending the time series, extracting the power spectra via a Fast Fourier Transform (FFT), and then computing the sum of frequencies in the low frequency band (0.01–0.08 Hz). Quadratic detrending was not performed as the removal of filters below 0.01 Hz is a non-linear detrending. The ALFF measure at each voxel is the averaged square root of the power in the 0.01–0.08 Hz window, normalized by the mean within-brain ALFF value for that subject. See Equation 1 below, in which *i* is a within-brain voxel, *A*_*i*_ is the averaged square root of the power in the 0.01–0.08 Hz window in that voxel, *n* is the number of voxels within the brain; the Alff_*i*_ measure which is used in subsequent analyses is *A*_*i*_ normalized by the mean within-brain ALFF value for that subject. For fALFF, the measure *A*_*i*_ was scaled by total power across all available frequencies. All ALFF and fALFF images were then smoothed by a 8 mm FWHM 3D Gaussian kernel.

(1) $$Ai=\text{average}\sqrt{\text{power in 0}.\text{01 to 0}.\text{08 Hz},}\text{ and }{\text{Alff}}_{i}=\frac{Ai}{\frac{1}{n}\sum_{1}^{n}Ai}$$

### Statistical analysis methods

#### Paired *t*-test of original vs. regressed images

The effect of the motion and physiological noise regression on the ALFF values was assessed using a paired *t*-test comparing images with and without regression across all subjects. A significance threshold of 0.05 corrected for multiple comparisons using the false discovery rate (FDR) was used in all analyses.

#### GLM analyses

ALFF and fALFF with and without regression were predicted with diagnosis and site as factors, age as a covariate, and the diagnosis by age interaction using a general linear model (GLM) in SPM5. Preliminary analyses had shown no effects of sex or site by diagnosis interactions so these terms were eliminated in the final analysis. All significance thresholds were set to *p* < 0.05 FDR-corrected and an extent threshold of 10 voxels. Results were localized by transforming the MNI coordinates to Talairach using mni2tal (http://imaging.mrc-cbu.cam.ac.uk/imaging/MniTalairach), then using the Talairach Client (Lancaster et al., 2000) and confirming the results visually using the atlases included with Mricron (http://www.mccauslandcenter.sc.edu/mricro/mricron/main.html).

#### Meta-analysis

The analyses described above analyzed all the data together, as a “mega-analysis.” We also performed SZ > HC and HC > SZ contrasts for each site separately, in order to assess the replication of the effects from the entire dataset within smaller subsets. This allowed us to perform an immediate meta-analysis. Each site's results as statistical t-maps were converted to Cohen's d effect size maps (Cohen, 1992); the weighted mean of the Cohen's d effect sizes across sites is a meta-analytic effect size image, which we compared to the effects found in the original, mega-analysis. If the meta-analytic effect sizes are larger than the mega-analysis effect sizes, then we have lost power by pooling the subjects across sites. If the mega-analysis effect sizes are similar or larger, the pooling across sites and including site as a factor is sufficient to account for site differences.

## Results

### Subject characteristics

A total of 306 subjects (147 SZ, 159 HC) had resting state fMRI data that passed quality assurance criteria. There was no significant effect of diagnosis on SFNR in the subjects in the overall, retained sample (*p* > 0.83) or within any site. The maximum translation did not differ with diagnosis (*p* > 0.86), nor did average root mean square translation (*p* > 0.09). SZ showed slightly greater mean framewise displacement (absolute sum of scan to scan movement) only in two sites, represented here as sites 2 (*p* > 0.024) and 5 (*p* > 0.01). Six subjects were excluded because they withdrew from the study; of the 70 subjects whose data were not suitable for inclusion, 44 were patients and 22 were controls, 47 (71%) were male, and their mean age was 41.5 years old. The two sites with the highest number of rejected data sets were both Siemens sites, with 21 subjects and 10 subjects rejected; both these sites began recruitment early and continued late attempting to collect more subjects, and recruited a number of patients who were unable to lie still in the scanner. The GE and two other Siemens sites each had 9 datasets rejected, while the two sites with the fewest rejected datasets had 5 and 3 total.

Patients and controls in the final sample were demographically similar across sites (Table 1) and patient symptom severity was comparable across sites (Table 2), though some demographic difference between sites were present. While the number of women and men participants were not balanced, the distribution across diagnoses and sites were not significantly different by a chi-square test (*p* > 0.05). A Two-Way Analysis of Variance (ANOVA) with site and diagnostic category as factors showed the mean ages did not significantly differ between SZ and HC, but site 3 had significantly older subjects overall than did the other sites [with the exception of site 2; *F*_(6, 291)_ = 4.3, *p* < 0.0001]. The HC group tended to be college graduates (SES code 2), whereas the SZ group had only “some college (>1 year)” (SES code 3), except for sites 2 and 3 where the median response for the SZ group was “high school graduate.”

**Table 1:** **Subject numbers, gender, age, and median education by data collection site**.

**Site code**	**Total *N***	**Sz**	**Sz M/F**	**Sz mean age (std.dev)**	**Median SES education (self)**	**HC**	**HC M/F**	**HC mean age (std.dev)**	**Median SES education (self)**
1	49	22	18/4	34.4 (8.8)	3	27	20/7	34.4 (9.3)	2
2	18	9	8/1	41.1 (12.5)	4	9	6/3	39.3 (9.1)	3
3	51	24	20/4	44.8 (12.1)	4	27	21/6	42.5 (12.6)	2
4	51	23	19/4	34.9 (11.9)	3	28	21/7	35.6 (11.6)	3
5	27	13	9/4	36.7 (10.7)	3	14	10/4	36.4 (9.1)	2
6	57	29	17/12	36.3 (11.1)	3	28	16/12	34.6 (10.6)	2
7	53	26	20/6	39.0 (11.7)	3	27	20/7	36.3 (10.3)	2

**Table 2:** **Disease duration, PANSS total, positive, negative, and general scores by mean (range)**.

**Site code**	**Duration of illness in years (range)**	**Total PANSS (range)**	**PANSS pos (range)**	**PANSS neg (range)**	**PANSS general (range)**
1	13.1 (2–27)	60.2 (33–103)	15.9 (7–28)	15.4 (7–33)	28.8 (18–48)
2	21.0 (3–41)	58.9 (36–80)	15.4 (9–21)	15.1 (7–24)	28.4 (18–38)
3	24.1 (2–40)	64.7 (43–107)	16.5 (9–24)	16.7 (9–39)	31.4 (20–47)
4	13.0 (3–41)	55.2 (38–81)	13.9(7–22)	13.4 (7–24)	27.9 (19–42)
5	15.1 (2–27)	54.2 (32–87)	13.5 (7–22)	14.6 (7–29)	26.1 (16–43)
6	15.9 (2–48)	53.8 (37–80)	14.0 (7–28)	13.1 (7–26)	26.7 (18–46)
7	18.6 (1–39)	56.5 (38–81)	15.7 (10–29)	13.6 (7–30)	27.3 (16–40)

### ALFF analyses

#### Paired *t*-test

**Figure 1:**
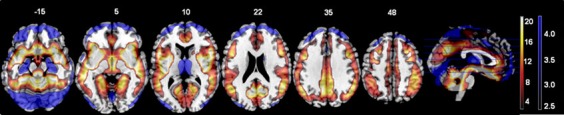
**The effects of motion and physiological signal regression on ALFF values: The regression step strongly increased ALFF values in more internal gray matter areas (shown in red to yellow, showing *t*-values from 2.5 to 20), and more weakly decreased ALFF values around the brain edges and thalamus (shown in blue, with *t*-values from 2.5 to 4.5).** All values from the paired *t*-test are thresholded at *t* > 2.5, FDR corrected *p* < 0.05. All images are in neurological convention (left is on the left) with the slice location on the z-axis shown.

The *t*-test comparing the original ALFF and regressed ALFF across all subjects showed the regression strongly increased ALFF values (*t* > 2, FDR corrected *p* < 0.05) throughout the interior of the cortex and brainstem, while it decreased the ALFF values around the external edges, in the frontal and occipital/cerebellar extremes, the inferior temporal cortices and thalamus (see Figure 1 below). The strongest effect was the increase in the right angular gyrus [51, −63, 36; *T*_(1, 317)_ = 25.77, *p* < 0.0001 FDR corrected].

#### GLM analyses

**Figure 2:**
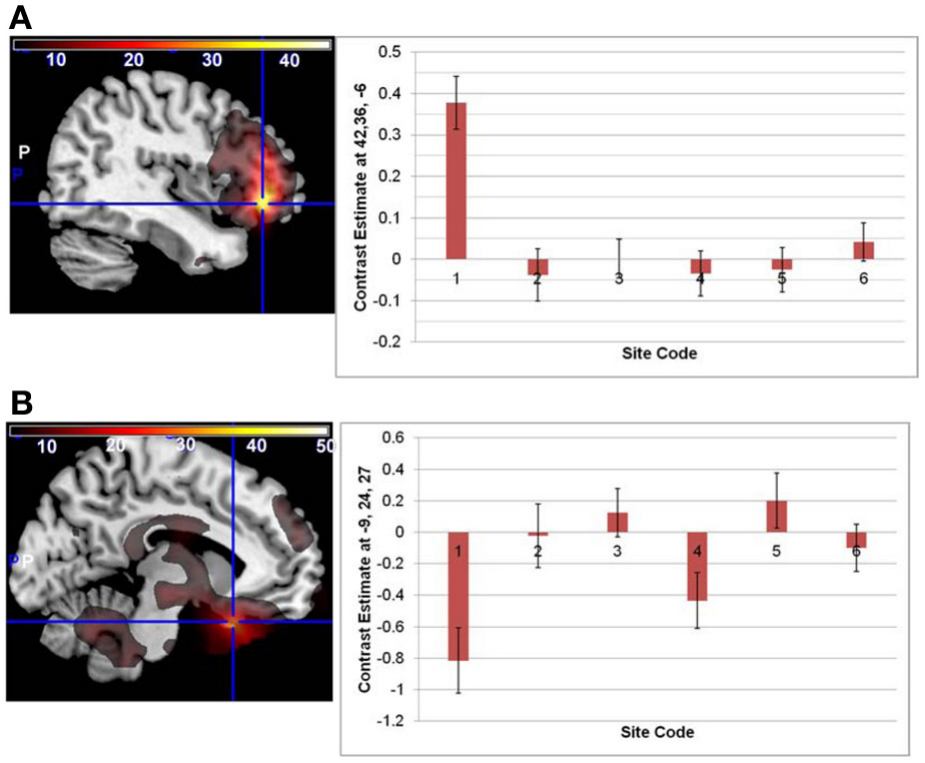
**The main effect of site in the original ALFF images, at the loci of maximal effect sizes (thresholded at *F* > 5; the color bar indicates the *F*-values). (A)** Left: F-map overlaid on canonical brain (MNI coordinates 42, 36, −6); Right: The effects for each site. Site 7, a 3T Siemens scanner, was the implicit baseline; the x-axis is the 6 other sites, with the GE site first. **(B)** Same but at MNI coordinates −9, 24, 27.

There were no significant interactions between the effects of age and diagnosis in the GLM analyses of either the original or regressed ALFF images. The main effect of site was found throughout the brain; the largest effects in the original ALFF measures were in the right inferior frontal cortex [42, 36, −6; *F*_(6, 291)_ = 34.97, *p* < 0.0001], and the bilateral gyrus rectus [−9, 24, −27; *F*_(6, 291)_ = 29.89, *p* < 0.0001]. These effects were largely due to the 3T GE (first column on the x-axis) being significantly different from other sites, with the other sites having smaller effects (Figure 2). Secondary analyses removing the 3T GE site were also conducted, using the 3T Siemens sites only. The effect of site was still significant in various cortical and subcortical areas; the maximal effect size was found near the loci of Figure 2B, as would be expected where the effects for those sites differ from zero. The effects of diagnosis discussed below were not affected by the removal of the GE site, other than through a reduction of the *t*-values as would be expected with the reduced number of subjects.

**Figure 3:**
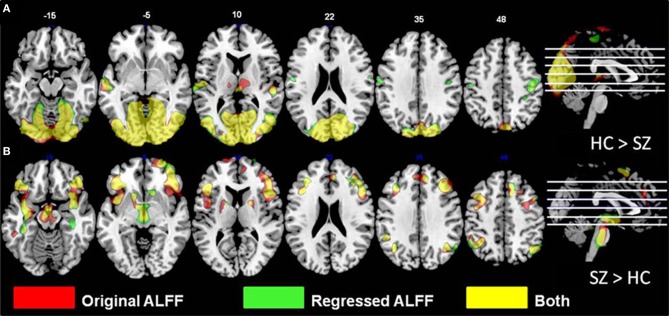
**The differences in ALFF values between diagnostic groups. (A)** Areas where healthy subjects had significantly larger values than subjects with schizophrenia, thresholded at *p* < 0.05 (FDR corrected; *t* > 2.6), for original and regressed ALFF (red and green, respectively). All suprathreshold voxels for a given contrast are shown with the same color. The areas in each cluster that are yellow are areas where the effect of diagnosis passes the significance threshold in both the original and motion-regressed ALFF data. **(B)** Areas where subjects with schizophrenia had greater values than healthy subjects, same thresholds and colors.

Overlays of the thresholded contrasts for the effects of diagnosis from the two analyses are shown in Figure 3, with the original ALFF results in red, the regressed ALFF effects in green, and yellow where the two both pass significance. HC showed significantly stronger ALFF measures than SZ did in the posterior occipital cortex, and into the superior parietal cortex, and this was not changed by the regression step (Figure 3A and Table 3A), except for areas of the thalamus and cerebellum which were significant in the original but not regressed images. The SZ group showed significantly greater ALFF measures than the HC group, in contrast, in medial and dorsal frontal regions, insula, and hippocampal/amygdala regions (Figure 3B and Table 3B). The SZ > HC effects in the original ALFF measures (in red) are stronger in the frontal cortex, and stronger in the subcortical and brainstem areas for the regressed ALFF (green), but overall the two analyses largely agree on the regions of significant group differences. The correlation between the *t*-values in these original and regressed ALFF contrasts was ρ = 0.97, with the original ALFF *t*-values in the HC > SZ contrast being higher on average across all within-brain voxels by 0.02. Table 3 shows that with the exception of the cerebellar and thalamic clusters, the ALFF significant clusters in the original analyses were identified in the regressed ALFF analysis.

**Table 3A:** **Areas where HC > SZ from the original ALFF analysis, in descending order of effect size**.

**Cluster no.**	**MNI coords**			**Cluster size**	**Voxel *p***	***Z*-score**	**Hemi-sphere**	**Region**	**Brodmann area**
1*	27	−84	33	6116	0	6.52	Right	Precuneus	19
	*18*	−*87*	*36*		*0*	*6.21*	*Right*	*Cuneus*	*19*
	*18*	−*69*	*12*		*0*	*5.61*	*Right*	*Posterior cingulate*	*31*
2*	−66	−18	6	162	0.004	3.7	Left	Superior temporal Gyrus	42
	−*57*	−*18*	*0*		*0.004*	*3.68*	*Left*	*Superior temporal Gyrus*	*22*
3*	−3	−18	78	352	0.003	3.77	Left	Medial frontal gyrus	6
	*3*	−*36*	*60*		*0.003*	*3.77*	*Right*	*Paracentral lobule*	*5*
	*24*	−*27*	*57*		*0.004*	*3.66*	*Right*	*Precentral gyrus*	*4*
4*	57	−21	6	60	0.004	3.74	Right	Superior temporal gyrus	41
	*63*	−*15*	*3*		*0.007*	*3.46*	*Right*	*Superior temporal gyrus*	*22*
5*	−60	−66	−27	36	0.008	3.46	Left	Cerebellum, posterior lobe	Declive
6*	39	−30	60	31	0.018	3.13	Right	Postcentral gyrus	3
7	54	−69	−27	23	0.018	3.12	Right	Cerebellum, posterior lobe	Declive
8	12	−12	3	11	0.021	3.07	Right	Thalamus	Ventral lateral nucleus
9*	54	−12	42	14	0.024	3	Right	Precentral gyrus	4
	*45*	−*12*	*45*		*0.035*	*2.84*	*Right*	*Precentral gyrus*	*6*

**Table 3B:** **As in 3A but for the SZ > HC contrast in the original ALFF analysis**.

**Cluster no.**	**MNI coords**			**Cluster size**	**Voxel *p***	***Z*-score**	**Hemi-sphere**	**Region**	**Brodmann area**
1^*^	−42	36	−3	202	0.022	4.78	Left	Middle frontal gyrus	47, 46
	−*45*	*30*	*6*		*0.022*	*4.41*	*Left*	*Inferior frontal gyrus*	*45*
2	−24	−42	−42	87	0.022	4.09	Left	Cerebellar tonsil, gray matter	
3^*^	51	18	15	193	0.022	4.57	Right	Inferior frontal gyrus	45, 13
	*45*	*36*	−*9*		*0.022*	*4.31*	*Right*	*Middle frontal gyrus*	*11*
4^*^	−51	−6	−45	145	0.022	4.49	Left	Inferior temporal gyrus	20
5^*^	27	69	−3	98	0.022	4.21	Right	Superior frontal gyrus	10
6^*^	−51	−24	−33	97	0.022	4.12	Left	Fusiform gyrus, uncus	20
7^*^	−30	−9	−15	49	0.022	4.08	Left	Amygdala, hippocampus	
8^*^	−3	−6	−12	38	0.022	4.05	Left	Hypothalamus	
9^*^	6	42	27	29	0.022	3.92	Right	Medial frontal gyrus	9
10^*^	−42	3	9	12	0.022	3.91	Left	Insula	48
11^*^	−39	9	−9	17	0.023	3.85	Left	Insula	48
12^*^	−18	72	9	27	0.028	3.67	Left	Superior frontal gyrus	10
13^*^	18	3	9	11	0.028	3.65	Right	Putamen	

*Maximal voxels are shown first, with secondary maxima in the same cluster shown in italics. Voxel p = FDR corrected p-value*.

* *Indicates a cluster which was matched in the regressed ALFF analysis*.

#### Meta- vs. mega-analysis

**Figure 4:**
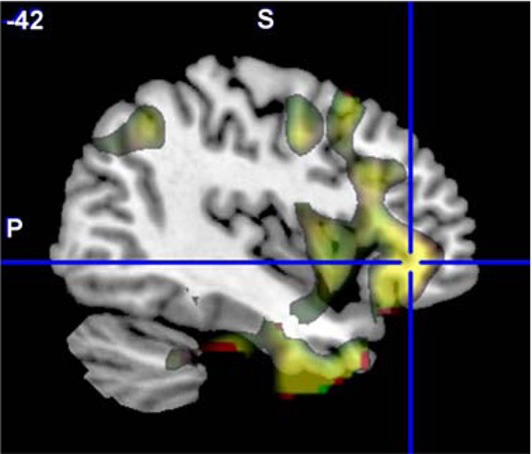
**The original ALFF mega-analysis Cohen's d image for SZ > HC (in red) and the meta-analysis weighted Cohen's d image (in green) overlaid on a template brain.** Both analyses are thresholded at *d* = 0.3 (moderate effect size); areas in yellow indicate both analyses passed the threshold. The cross hair is at (−42, 36, −3), the area of maximal significance in the original ALFF mega-analysis for SZ > HC.

The original ALFF data from each site were separately analyzed with a GLM including diagnosis as a factor, age as a covariate, and the age × diagnosis interaction. The SZ > HC and HC > SZ contrasts were calculated for each, and a weighted Cohen's d image to correct for differences in between-site samples sizes was calculated over all seven sites for each contrast. This weighted Cohen's d image can be compared directly to the Cohen's d image from the original, mega-analysis which included site as an additional factor (see Figure 4 below, with the mega-analysis results in red, the meta-analysis results in green, and the overlap in yellow). The largest effect size for SZ > HC in the original data was in the left middle frontal gyrus into the inferior triangular cortex (−42, 36, −3; *d* = 0.69 and 0.72 in the mega- and meta-analysis respectively). For the HC > SZ contrast, the maximal result at 27, −84, 33 in the right precuneus had an effect size of 0.76 in the mega-analysis and 0.77 at the same location in the meta-analysis.

We created masks for right and left BA 17 and 19 using the WFU Pickatlas (Maldjian et al., 2003), to capture the effects where HC > SZ, and frontal inferior triangular cortex for SZ > HC. Table 5 at the end of the Results section includes the mean Cohen's d for the HC > SZ contrast for BA 17 and 19 for the mega analysis, the weighted meta-analysis, and the range of values across the different sites, for ALFF, fALFF, and the regressed fALFF; and the same values for the SZ > HC contrast in the frontal inferior triangular region, for ALFF and regressed ALFF only. SZ > HC results for fALFF were not calculated since nothing passed significance in the mega-analysis.

### fALFF analyses

#### Paired *t*-test

**Figure 5:**
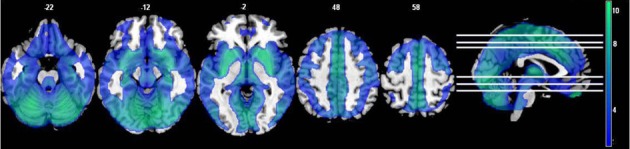
**The effects of motion and physiological signal regression on fALFF: The regression step decreased fALFF values throughout most of the gray matter and subcortical areas (*p* < 0.05, FDR corrected; *t* > 1.7; color bar indicates the *t*-value range, and slice locations are indicated)**.

The fALFF values from the original and regressed fMRI data showed significant differences throughout the cortex. The regressed images had lower fALFF values in the paired *t*-test (see Figure 5), with the strongest differences being throughout the midline and subcortical regions.

#### GLM analyses

**Figure 6:**
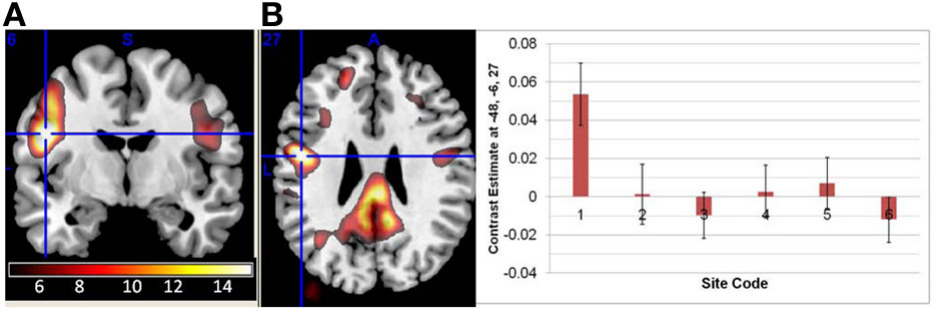
**The main effect of site in the fALFF analysis. (A)** The effect of site in the original fALFF analyses, thresholded at *F* > 5 and overlaid on a template brain. Cross hair at the maximal voxel (−48, −6, 27). **(B)** The contrast estimates across the different sites, with the first site being the 3T GE and the others being 3T Siemens scanners. Site 7 was the implicit baseline.

In both the original and regressed fALFF measures, there were significant effects of both site and diagnosis, but no significant age × diagnosis interactions. In the original fALFF analysis, the effect of site covered almost the entire brain; the largest contiguous cluster was over 70,000 voxels. The strongest effect of site in the original fALFF analysis was in the left precentral gyrus at −48, −6, 27 [*F*_(6, 296)_ = 11.6, *p* < 0.00001 FDR corrected], and again was due to the GE site being the most different from the other sites, as shown in Figure 6. In the motion regressed fALFF analysis, the maximal effect was in the posterior/middle cingulate at 3, −30, 27 [*F*_(6, 296)_ = 10.6, *p* < 0.00001 FDR corrected], with a similar pattern of effects across the sites. The secondary analysis on the Siemens sites' data only, in both the fALFF analyses, showed no significant site effects. However, the effects of diagnosis as reported below were again not affected other than through a reduction in the maximal *t*-values.

**Figure 7:**
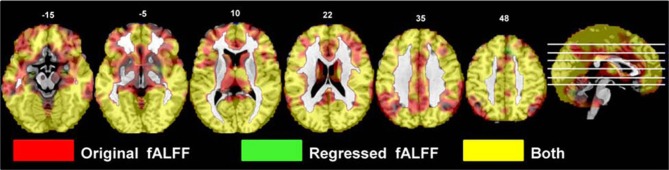
**The differences in fALFF values between diagnostic groups.** Areas where healthy subject had significantly larger fALFF values than did subjects with schizophrenia, thresholded at *p* < 0.05 (FDR corrected; *t* > 1.7) for the original and regressed fALFF (red and green, respectively). All suprathreshold voxels for a contrast are shown in the same color. The areas in each cluster that are yellow are areas where the effect of diagnosis passes the significance threshold in both the original and regressed fALFF data.

The areas where HC > SZ reached significance were throughout cortical gray matter in both analyses, though the effects were more wide spread in the original fALFF measures, particularly in the frontal and midline cortex. The maximal voxels and clusters for HC > SZ in the original fALFF results are listed in Table 4. In Figure 7 the HC > SZ statistical map with a threshold of FDR-corrected *p* < 0.05 is shown in red for the original fALFF analysis, green for the regressed fALFF analysis, and yellow where the two overlap. The correlation between the *t*-values in these original and regressed fALFF contrasts was ρ = 0.96, with the original fALFF *t*-values in the HC > SZ contrast being higher on average across all voxels by 0.5. Table 4 and Figure 7 show that this increased mean *t*-value did not translate to a change in the significant effects.

**Table 4:** **Areas where HC > SZ from the original fALFF analysis, in descending order of effect size**.

**Cluster no.**	**MNI coords**			**Cluster size**	**Voxel *p***	***Z*-score**	**Hemi-sphere**	**Region**	**Brodmann area**
1^*^	21	−84	30	70203	0	6.32	Right	Cuneus	19
	*51*	−*69*	*0*		*0*	*6.28*	*Right*	*Middle temporal gyrus*	*37*
	*15*	−*69*	*12*		*0*	*6.12*	*Right*	*Posterior cingulate*	*31*

*Maximal voxels are shown first, with secondary maxima in the same cluster shown in italics. Voxel p = FDR corrected p-value*.

* *Indicates a cluster which was matched in the regressed fALFF analysis*.

#### Meta- vs. mega-analysis

**Figure 8:**
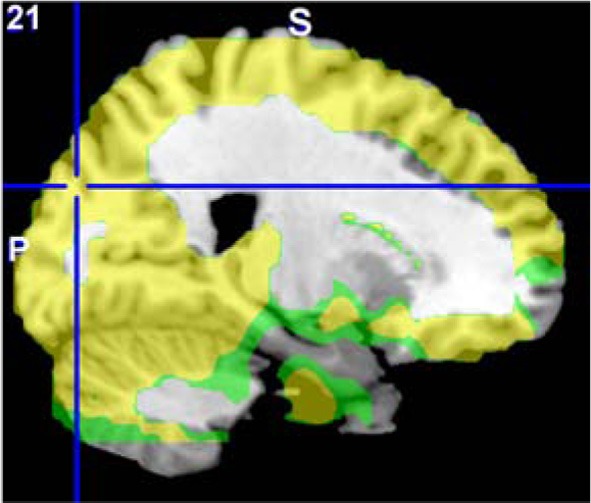
**The original fALFF mega-analysis Cohen's d image for HC > SZ (in red) and the meta-analysis weighted Cohen's d image (in green) overlain on a template brain.** Both analyses are thresholded at *d* = 0.3 (moderate effect size); areas in yellow indicate both analyses passed the threshold. The cross hair is at (21, −84, 30) the area of maximal significance in the original fALFF mega-analysis for HC > SZ.

The original fALFF data from each site were separately analyzed with a GLM including diagnosis as a factor, age as a covariate, and the age × diagnosis interaction. The HC > SZ contrasts were calculated for each, and a weighted Cohen's d image was calculated over all seven sites as in the ALFF analysis. This weighted Cohen's d image can be compared directly to the Cohen's d image from the original, mega-analysis which included site as an additional factor (see Figure 8). For the HC > SZ contrast, the maximal result at 21, −84, 30 in the right cuneus had an effect size of 1.07 in the mega-analysis and 1.22 at the same location in the meta-analysis.

The same mask for BA 17 and 19 for the HC > SZ contrast was used on the fALFF and regressed fALFF analyses from the mega-analysis and each site separately, to calculate a mean Cohen's d from each site, from the mega-analysis, and a weighted Cohen's d as meta-analysis. The weighted Cohen's d was consistently slightly greater than the mega-analysis effect size, from 3% greater in the ALFF analysis of HC > SZ, to 21% larger in the fALFF analysis. Table 5 lists the means across various contrasts and analyses, as well as the minimum and maximum Cohen's d found in the individual site analyses.

**Table 5:** **Comparison of the mean Cohen's d from the mega-analysis and from the meta-analysis, for selected regions of interest in the original ALFF, fALFF and regressed fALFF(R-fALFF), separately for HC > SZ and SZ > HC analyses**.

**Analysis**	**Mega**	**Meta**	**Range of effect sizes from each site**
**Mean Cohen**'**s d from HC > SZ, Brodmann areas 17+19**			
ALFF	0.36	0.38	0.12–0.63
R-ALFF	0.38	0.40	0.10–0.63
fALFF	0.72	0.87	0.41–1.01
R-fALFF	0.65	0.67	0.30–1.02
**Mean Cohen**'**s d from SZ > HC, frontal inferior triangular cortex**			
ALFF	0.26	0.27	−0.02–0.46
R-ALFF	0.24	0.25	0.02–0.50

*SZ > HC for fALFF and R-fALFF are not included as no significant effects were found in the mega-analysis*.

## Discussion

We report the findings of voxel-wise ALFF and fALFF analyses, with and without regression of commonly-used nuisance variables, in a multi-site study of several hundred subjects with SZ and healthy subjects. The goal of this research was twofold. First, to use a large, multi-site sample to confirm the differences in these resting state measures between patients with SZ and controls. The second purpose was to identify whether choice to regress head movement and physiological signals makes a difference, not just in the ALFF or fALFF values *per se*—i.e., removing contaminants and getting a “truer” value of ALFF for an individual—but in drawing conclusions about cases and controls when we are studying SZ. Healthy subjects show greater ALFF and fALFF in the occipital and parietal cortex than do the patients, and particularly in the fALFF analyses this is a moderate to strong effect size even with the variability across data collection sites. The subjects with SZ show greater ALFF in the frontal cortex, but the effect size is small and quite variable across data collection sites, with or without motion and physiological noise regression. Both ALFF and fALFF measures are quite robust to the effects of scanner type, with the same pattern of diagnosis differences identified with either the mixed-scanner group or the subset of consistent scanners. The effect of regression is quite strong on the ALFF and fALFF values for individuals, but has no notable effect on the contrasts between patients and controls.

### Resting state ALFF and fALFF in schizophrenia

**Figure 9:**
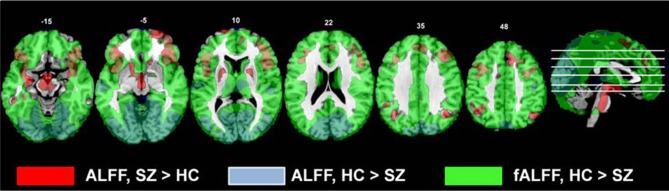
**The comparison of the original ALFF and fALFF findings for the HC > SZ and SZ vs. HC contrasts.** All contrasts are thresholded at *t* > 2.5; the original ALFF SZ > HC results are in red, the original ALFF HC > SZ results are blue, and the original fALFF HC > SZ results are in green.

In these data, the effect of scaling ALFF by the total power across available frequencies in the fALFF analysis was to strengthen group differences in the areas where healthy subjects showed greater amplitudes, identifying that the healthy subjects had greater fALFF across the cortex. The HC > SZ ALFF results were subsumed within the fALFF results, but the SZ > HC were not (Figure 9). Thus both ALFF and fALFF analyses need to be performed, to get a clearer picture of the pattern of results.

Our findings are in contrast with the previous findings of greater fALFF measures in subjects with SZ, but in keeping with numerous papers performing spectral analyses on timecourses from ICA. Comparing approximately 30 patients with SZ to 30 HC, Hoptman et al. (2010) found controls showed greater ALFF and fALFF in the posterior brain as in our results, but they also found patients showed greater fALFF in the parahippocampal gyrus, medial prefrontal cortex, anterior cingulate, and caudate. Since their subject population had a similar mean age, duration of illness, and other characteristics as the overall FBIRN sample, the inconsistent results are not likely due to sample differences. Spectral analyses of time courses from ICA methods, however, have consistently shown that healthy subjects have greater power in the lower spectral frequencies (usually below 0.08 Hz) than do patients with SZ, while the patients with SZ have greater power in the spectral frequency bins above 0.08 Hz. This has been observed in analyses of auditory oddball data (Garrity et al., 2007; Calhoun et al., 2008a,b, 2012; Kim et al., 2009b), as well as resting state data (Calhoun et al., 2008a; Damaraju et al., in preparation). In each of these studies the time courses were normalized or z-scored prior to the spectral analysis, so that fluctuations were expressed as standard deviations from the mean; in the current ALFF analysis the time courses were detrended but not otherwise scaled. According to the discrete version of Parseval's theorem (Plancherel's theorem), the variance of a time series is equivalent to the summed power over all frequencies. Normalizing the time course divides by its standard deviation, and thus effectively expresses the power in each spectral bin relative to the summed power over all frequencies; therefore these published analyses were performing an analysis of the spectra from ICA timecourses which in the lowest frequency bin value is very similar to fALFF. The finding in the FBIRN data that fALFF is stronger in healthy subjects throughout the cortex is in keeping with these previous findings.

In Zuo et al.'s (2010) analyses of healthy subjects, z-scored ALFF was significantly stronger than z-scored fALFF in the subcortex and boundary areas of the temporal lobe, and thus likely to reflect vascular and pulsatile effects. We did not find that subjects with SZ show greater ALFF than the healthy subjects in these areas, and our findings in other regions remain significant when head movements and WM/CSF noise is regressed from the temporal signals; thus the pattern of greater ALFF in the frontal areas in the patient group vs. the HC is not likely due to vascular or head movement differences.

Never-treated first-episode SZ have shown smaller ALFF values in ventral medial frontal regions at baseline, and shown greater ALFF relative to healthy subjects after 6 weeks of treatment in the right and left putamen (Lui et al., 2010). Moreover, with 60+ of medication naïve patients and an equal number of HC, Huang et al. (2010) found patients showed decreased ALFF in orbital, polar, frontal medial cortex, and greater ALFF in the putamen. In our much larger sample of chronic, medicated patients, we find greater ALFF in the putamen, as well as in many areas of the frontal cortex. We did not observe any frontal cortical areas where the patients had lower ALFF values than the controls. Lui et al. identified areas where ALFF increased in patients with 6 weeks of treatment (see their Table 3); we identified whether the reported voxels lay within our weighted Cohen's d-map of the ALFF results for SZ > HC (with an effect size >0.3). Of the nine areas reported as increasing with treatment by Lui et al., all but three overlapped with our SZ > HC results. The overlap between the Lui findings and the increased frontal ALFF in our analyses as well as those of Yu et al. (2012) suggests that the development of increased ALFF in some regions of the frontal cortex may be possibly an interaction between disease progression and age, or a long-term medication effect.

The increased ALFF measures for patients outside of the frontal and inferior temporal lobes included the left insula, amygdala, hippocampus, hypothalamus, cerebellar tonsil, and right putamen. Hoptman et al. (2010) also found increases in ALFF in the left hippocampus. The hippocampus has been consistently identified as problematic in SZ as well as other disorders (Small et al., 2011), and the hippocampal-frontal dysfunctional connectivity implicated in SZ (Godsil et al., 2013) has been linked to cognitive and emotional dysfunction. In the insula, Yu et al. (2012) reported a cluster where controls showed greater ALFF than patients, which seems to be a discrepancy with our results; but a closer comparison of their coordinates and our findings show their results are very close to the cluster of increased ALFF in controls that we identified in the superior temporal lobe, whereas our insular results are more anterior. The insula has been linked with psychotic symptoms in depression (Busatto, 2013), and increased ALFF in the left insula has also been found in bipolar disorder (Liu et al., 2012); thus while the frontal and hippocampal differences may relate to the executive function disorder, the insular dysfunction may be reflective of the psychosis in general.

The occipital, superior parietal lobe and precuneus areas consistently showed both lower ALFF and fALFF measures in SZ. The occipital lobe finding is in keeping with Hoptman et al's hypothesis that these low frequency fluctuation reductions in SZ are in the primary sensory areas, with downstream ramifications. The precuneus in particular has been implicated in SZ DMN dysfunction in both resting state and task-based analyses (Garrity et al., 2007; Calhoun et al., 2012). In addition, in healthy subjects, (Zou et al., 2012) examined the relationship between resting state ALFF and N-back working memory task performance and associated BOLD signal. In the more difficult memory conditions, they found positive correlations between resting state ALFF measures and task-related activation in the superior posterior parietal lobe and precuneus; they found negative correlations in the medial frontal and bilateral superior temporal lobe. The resting state ALFF measure in the parietal lobe/precuneus region was significantly positively correlated with behavioral performance in the hardest memory condition. Patients with SZ in general have working memory performance deficits; they show smaller BOLD signal increases most often in the dorsolateral prefrontal cortex during an N-back task (Callicott et al., 1998), or overactivity to achieve the same performance levels (Brown et al., 2009; Potkin et al., 2009); but they also show dysfunctional activations and connectivity in the parietal lobe while performing other working memory tasks (Kim et al., 2009a, 2010). Our findings support Zuo et al.'s interpretation that a network of fronto-parietal areas is required for working memory function, and speculate that the lower resting state ALFF and fALFF in the parietal lobe is a marker for a more general underlying dysfunction which makes activation of the working memory network more difficult. Functional connectivity approaches both using ICA or other methods have also identified widespread weaker resting state cortico-cortical connectivity in patients with SZ than controls (Lynall et al., 2010; Yu et al., 2013), suggesting the fALFF measures relate to the capacity for broader network coordination. Lower resting state measures need not be specific to working memory dysfunction, but could be a more general marker for disconnectivity or disorganized circuitry which could be expressed in many cognitive deficits. More specific studies are required to assess if deficits in resting state ALFF are a predictor of cognitive or perceptual dysfunction in SZ.

### Aggregating voxel-wise analyses of resting state fMRI over multiple scanners

There were significant and pervasive differences between the sites in ALFF and fALFF measures in various brain regions. There were, however, no significant interactions between diagnosis and site, when we included that term in the model.

The effects of motion and CSF/WM regression on the ALFF and fALFF measures were robust. Regression effects on ALFF vary by brain region, increasing ALFF in the center and decreasing around the brain edges; the regression does not simply reduce power in the higher temporal frequencies on a voxel-wise basis across the brain. In contrast, the regression step reduces the fALFF values throughout the brain. In exploring this effect more closely, we examined the gray matter time courses of a random selection of subjects across sites, to determine the effects of regression on both the 0.01–0.08 Hz amplitudes and the 0.09–0.25 Hz amplitudes. The effect of regression was to reduce the lower frequencies more than the higher frequencies, and thus the ratio computed for fALFF was reduced. This is not unexpected, given the aliasing of the cardiac and respiratory signals into the lower frequencies (Lund et al., 2006; Birn et al., 2008). Birn et al. (2008) in particular showed that with a 2s TR, the sampled time course for respiration has most of its power below 0.05 Hz, and thus would reduce the fALFF measures. However, the regression effects were similar across subjects, so that the effect on the contrast between patients and controls was minimal and the sensitivity to disease-specific effects did not change. The pattern of significant clusters were similar (see Figures 3, 7), and the effect of regression on the mega-analysis and meta-analysis effect sizes in ALFF and fALFF was inconsistent (Table 3). The regression step did not generically reduce variability and increase sensitivity to between-group differences, or change the overall pattern of results.

While the fMRI protocols were developed to be as similar as possible across the scanners, with slice-selection parameters and multi-coil combination techniques the same across site (Glover et al., 2012), and head movement and SFNR measures across sites were similar, the effect of site on the contrast between subject groups could not be completely eliminated. The site effects were more pronounced in the ALFF analyses, with differences between the Siemens sites remaining significant throughout the gray matter even after removing the GE data. The GE scanner did produce images with a higher mean value overall, which means variation around that mean as measured in ALFF could be increased; however, given we were scaling the raw ALFF by the mean, this effect should be reduced. To confirm this, we also performed intensity normalization on the images prior to ALFF analysis, reducing the difference in mean values, and the effect of site did not change. Robust differences between GE and Siemens fMRI data have been found previously using other measures (Friedman et al., 2008; Greve et al., 2011; Glover et al., 2012). A previous analysis of SFNR measures in some of these scanners (two Siemens 3T scanners, this GE 3T and another GE 3T scanner) showed the GEs consistently showed higher background SFNR measures, though the physiologically based, signal-weighted SFNR was consistent across sites, suggesting again that we should not see these inter-site differences (Greve et al., 2011). That analysis, however, did not look at cortex regionally, and explicitly ignored areas which are subject to B0 distortion, such as the orbital and inferior temporal cortex where the GE effects in ALFF were the strongest, as in Figure 2. The strongest effects of site (*F* > 15) were in the frontal inferior orbital areas, as well as the more medial gyrus rectus, well into the areas of B0 disortion. Given this pattern, together with the standardization of imaging protocols, slice selection profiles, and the combination algorithm for multiple coils, we conclude that the primary source of the remaining GE/Siemens differences is likely to be regional B0 distortion differences.

The inter-site differences in fALFF were not significant for the consistent subset of sites using the same scanner make and model. The variance normalization done as part of fALFF calculations seems to reduce those inter-site differences within a single make and model. While this might on the face of it seem to argue for (a) using fALFF rather than ALFF and (b) using a consistent make and model of scanner in a multi-site study, the fact of the matter is that the important differences between patients with SZ and HC were not affected by the inclusion of an outlier site. This supports the notion that both fALFF and ALFF effects are robust to make and model differences, and we continue to recommend including a site factor in any analysis model, as recommended by many others (Fennema-Notestine et al., 2007; Pardoe et al., 2008; Stonnington et al., 2008; Yendiki et al., 2010).

The meta-analysis of diagnosis effects, in which the analysis was performed for each site separately and then combined in a weighted Cohen's d, consistently gave a slight but consistently larger effect size than in the mega-analysis. Aggregating resting state datasets across multiple scanners in a mega-analysis carries with it a small penalty, reducing the differences between subject groups in this case, even when the scanner effects are included in the model. The implications for large-scale aggregated data analyses such as those needed for imaging genetics or clinical trials are supportive of including approaches such as that used by ENIGMA (Stein et al., 2012), in which each dataset is analyzed separately and the results combined in a meta-analysis. Both mega-analysis and meta-analysis techniques can identify very similar results, though there may be experimental designs that require one approach or the other. The value of these large-scale studies, however, is at least in part that the individual site, 30-subject analyses were not consistently indicative of ALFF differences in SZ. Thus doing a single small study would lead to misrepresentative results from an underpowered design, and doing multiple small-scale studies which could then be collected for a meta-analysis would not be likely to happen. In addition, with the large-scale samples we can explore relationships with clinical symptoms more thoroughly; initial analyses have indicated ALFF relationships with cognitive deficits in the schizophrenic population (Brandel et al., 2013). With these large-scale data sets collected prospectively to be as similar as possible, the mega-analysis results are definitive.

## Conclusions

In the analysis of patients with SZ and HC, the additional step of regressing out head movement parameters along with WM and CSF mean signals has no effect on the pattern of differences in ALFF and fALFF between diagnostic groups; thus the choice to do this regression or not should be based on other considerations, such as ease of pre-processing or what else the resting state measures are being used for. In combining multi-site resting state fMRI datasets across multiple scanners of the same make and model, which have already been standardized to the greatest extent possible, variance normalization of the timeseries data as is performed implicitly in fALFF can greatly reduce inter-site variation. However, it does not remove the site effects of different scanner manufacturers. And without that normalization, as in ALFF analyses, the site effects can be quite prominent. In a multi-site dataset analysis, a comparison of the mega-analysis results with the meta-analysis can help identify the extent to which site effects are being captured by including site as a covariate in the mega-analysis.

### Conflict of interest statement

The authors declare that the research was conducted in the absence of any commercial or financial relationships that could be construed as a potential conflict of interest.
